# Supplementary material for: High-potency ligands for DREADD imaging and activation in rodents and monkeys
Source: Nat Commun. 2019 Oct 11;10:4627. doi: 10.1038/s41467-019-12236-z (PMC6788984; doi:10.1038/s41467-019-12236-z)
Supplement: Supplementary file 1 — Supplementary Information [file 41467_2019_12236_MOESM1_ESM.docx]

**Supplementary Figures**

**Supplementary Figure 1.**

**The second-generation DREADD agonist C21 exhibits low in vitro DREADD affinity and potency compared to clozapine.**

We assessed Compound 21 (C21) and clozapine (**a**) binding in mouse and rhesus monkey (*Macaca mulatta*) brain sections expressing DREADDs. Low concentrations of [^3^H]clozapine bound to DREADDs in intact tissue sections in both species but [^3^H]C21 did not show any DREADD binding in the mouse (**b**) and showed only weak DREADD binding coupled with high off-target binding in the monkey (**c**). In DREADD-expressing HEK-293 membranes, C21 inhibited [^3^H]clozapine binding with low affinity (^hM3Dq^*K*_i_=230 nM; ^hM4Di^*K*_i_=91 nM) compared to clozapine (^hM3Dq^*K*_i_=3.5 nM; ^hM4Di^*K*_i_=2.8 nM) (**d**). In functional assays measuring excitatory hM3Dq DREADD activation in transfected HEK-293 cells, C21 elicited potent intracellular Ca^2+^ increases comparable to that of clozapine (**e**). However, C21 was less potent than clozapine at Gi/o activation in cells or tissue expressing hM4Di (**e-g**). Neither clozapine nor C21 produced detectable functional responses in cells (Supplementary Fig. 3) or tissues devoid of DREADD expression (**f**).

**
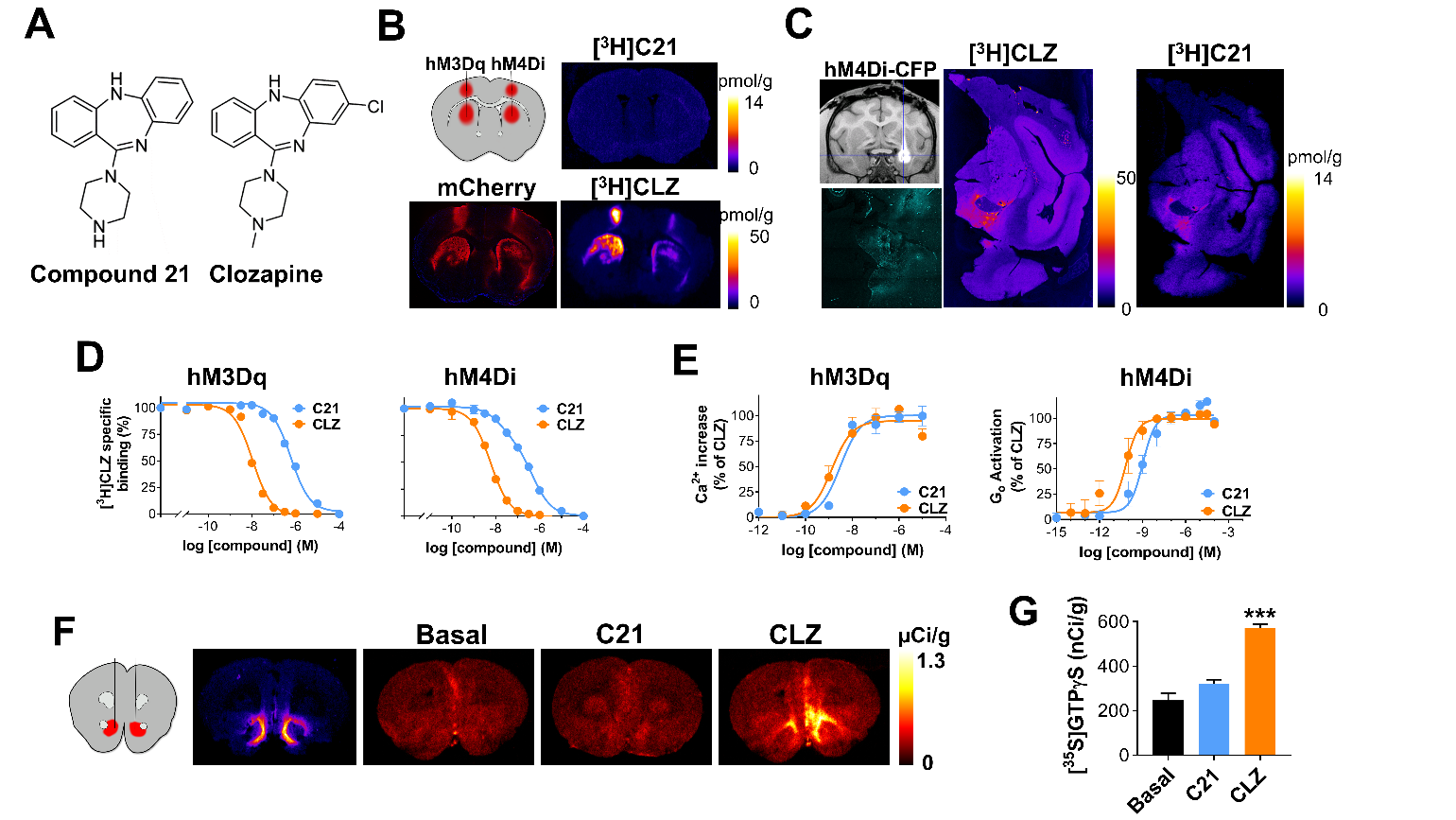
**

(**a**) Structures of C21 and clozapine (CLZ). (**b**) Unlike [^3^H]CLZ (3.5 nM), [^3^H]C21 (10 nM) does not bind to DREADDs expressed in mouse brain slices. (**c**) [^3^H]C21 (10 nM) and [^3^H]CLZ (3.5 nM) both bind to hM4Di expressed in the monkey amygdala but [^3^H]C21 exhibits lower selectivity. (**d**) Binding competition curves of [^3^H]CLZ versus increasing concentrations of C13 and C22 in HEK-293 cells expressing DREADDs. C21 has 100-fold less affinity than CLZ. (**e**) C21 and CLZ have comparable potency at inducing hM3Dq-mediated intracellular Ca2+ increases in HEK-293 cells but CLZ showed greater potency at hM4Di-dependent Go activation. (**f, g**) CLZ induces significantly greater hM4Di activation (measured as [^35^S]GTPγS binding) compared to C21 in rat brain sections. Data panels **d**, **e** and **g** represents mean ± SEM (error bars smaller than the data points are not displayed). In **g**, one-way ANOVA (F_(2,15)_=52.79) followed by Dunnett’s multiple comparison tests, *** represents p < 0.0001 compared with basal.

**Supplementary Figure 2.**

**C21 exhibits lower brain penetrance and weaker *in vivo* DREADD potency in rodents and in monkeys compared to clozapine**

As recently reported^4^, we found that systemic administration of C21 led to a low brain/serum ratios of the compound in mice and a similar result (CSF/serum) was observed in the monkey (**a, b**). These low ratios suggested poor brain penetrance. Unlike CNO^2,14^, C21 was not a substrate for the P-glycoprotein (P-gp) efflux pump (Supplementary Fig. 5). To assess direct brain engagement of DREADDs we injected wild-type (WT) and transgenic mice expressing hM3Dq or hM4Di in dopamine D1 receptor-expressing neurons (D1-hM3Dq or D1-hM4Di) intraperitoneally (IP) with [^3^H]C21 or [^3^H]clozapine and collected brain and organs 30 or 60 min later. [^3^H]C21 showed widespread uptake across various organs and in blood. The highest levels of [^3^H]C21 were observed in kidneys (Supplementary Fig. 3). In brain, [^3^H]C21 accumulated exclusively in ventricles without discernible DREADD engagement (**c-e**). In agreement with the results of the *ex vivo* autoradiography described immediately above, PET imaging revealed that 1 mg/kg C21 (mouse: IP, monkey: IV) led to low (~10%) *in vivo* DREADD occupancy in mice (**f, g**) and no measurable occupancy in the monkey (**h, i**). At 10 mg/kg, C21 achieved ~50% hM4Di occupancy in the monkey (**h, i**) but this dose also produced extensive displacement of [^11^C]clozapine binding at endogenous binding sites and non-specific effects, thus no further monkey studies were conducted.

C21 did not affect locomotor activity in WT mice at doses up to 1 mg/kg but, like in the monkey, produced non-specific effects at 10 mg/kg (**j**). In D1-hM3Dq mice, C21 exhibited a robust decrease of locomotor activity at 0.1 and 1 mg/kg (**k**) and seizure-like behavior at 10 mg/kg. Thus, no further experiments were performed at this dose. In D1-hM4Di mice, C21 produced a small but significant decrease in locomotor activity at 1 mg/kg and sedation similar to that observed in the other groups of mice at 10 mg/kg (**l**). The doses required to induce the same degree of DREADD-specific locomotor inhibition by clozapine were approximately 100-fold lower. DREADD-specific behavioral effects of C21 were also examined in transgenic rats expressing hM3Dq in tyrosine hydroxylase (TH)-expressing neurons in the ventral tegmental area (VTA) (**m**). Rats were injected IP with 1 or 5 mg/kg C21, which led to significant increases in locomotor activity, while clozapine produced comparable locomotor increases at a dose as low as 0.001 mg/kg (**n, o**). DREADD-mediated behavioral effects were observed at low levels of *in vivo* occupancy, likely due to supraphysiological viral-mediated DREADD expression and high intrinsic efficacy of C21 and clozapine.

In a competitive binding screen C21 showed a similar target profile to clozapine at 10 µM, but unlike clozapine, C21 exhibited binding to mu, kappa, and delta opioid receptors (Supplementary Fig. 8). C21 tested at 100 nM showed a similar profile to 10 nM of clozapine. However, given that C21 exhibited lower *in vivo* DREADD binding than clozapine, and that higher (~1000-fold) doses needed to drive DREADD-specific behaviors, this *in vitro* selectivity profile for endogenous targets does not appear to predict *in vivo* outcomes very well.

In PET studies using [^18^F]fluorodeoxyglucose (FDG) to measure changes in regional metabolic activity, we injected 1 mg/kg C21 (IP), a dose on the lower end of the range recently suggested for its use^4^. This dose produced significant changes in brain metabolic activity in WT mice (Supplementary Fig. 7), indicating that 1 mg/kg of C21 affects brain function and cannot be used for DREADD-assisted metabolic mapping (DREAMM) studies^8,9^, even though no behavioral effects were detected in our model at this dose. In contrast, an equipotent dose of clozapine (0.1 mg/kg) did not produce any significant changes in brain glucose metabolism (Supplementary Fig. 7).


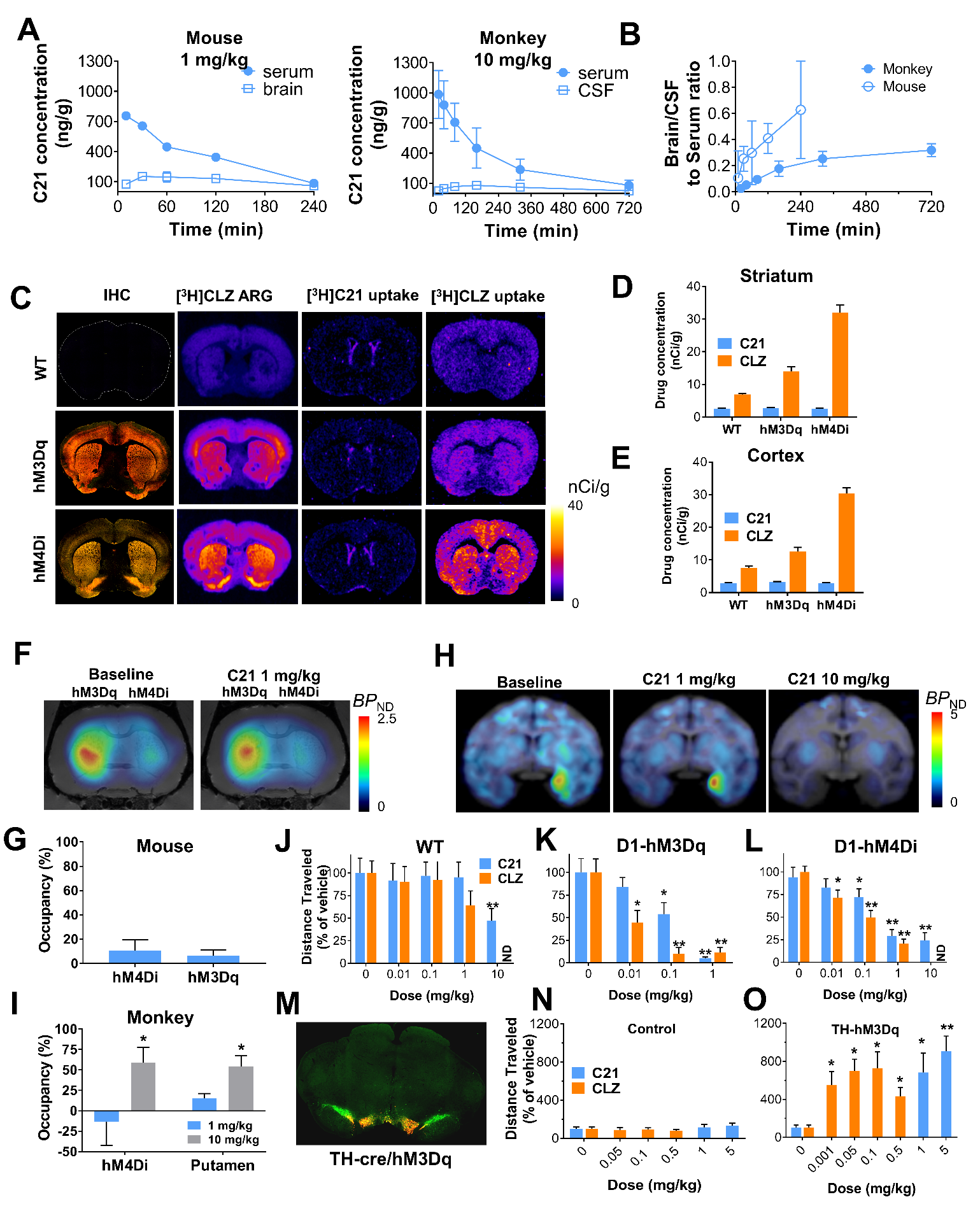


(**a**) Systemic C21 administration leads to low brain and high plasma concentrations in mice (n = 3 mice) and in monkeys (n = 2 monkeys) and (**b**) low brain/serum (mice) and CSF/serum ratios (monkey). (**c-e**) Immunohistochemical (IHC) and autoradiographic (ARG) localization of HA-DREADDs in transgenic mouse brain tissue (non-fused mCitrine (yellow) and fused HA-tag (red)) along with ex vivo [^3^H]C21 and [^3^H]CLZ brain uptake in striatum and cortex showing that [^3^H]C21 is not found on DREADD-expressing areas. Representative images from sections collected from 3 different mice per condition are displayed in **c**, quantification of the uptake in cortex and striatum is displayed in **d** and **e**. (**f, g**) C21 (1 mg/kg, IP) produces an approximate 10% displacement of [^11^C]CLZ in mice (n = 5 mice) expressing AAV-DREADDs in striatum. (**h, i**) Systemic delivery of 1 mg/kg C21 does not displace [^11^C]CLZ binding in monkeys (n = 2 monkeys) expressing AAV-hM4Di in the amygdala whereas 10 mg/kg displaces [^11^C]CLZ from both hM4Di and non-hM4Di expressing sites. (**j-l**) CLZ exhibits significantly greater *in vivo* potency than C21 in transgenic DREADD mice (n = 8 to 18 mice per condition) and (**m-o**) in transgenic rats (n = 10 rats per condition) expressing hM3Dq in tyrosine-hydroxylase (TH) neurons in ventral tegmental area (VTA). **m** is a representative IHC image (TH green, mCherry red) of the animals used in **o**. In all cases, data are represented as mean ± SEM. One or two-way repeated measures ANOVA followed by Dunnett’s multiple comparison tests were performed, * p < 0.05 and ** p < 0.01 compared with the respective vehicle.

**Supplementary Figure 3.**

**Biodistribution of C21 and C13.**


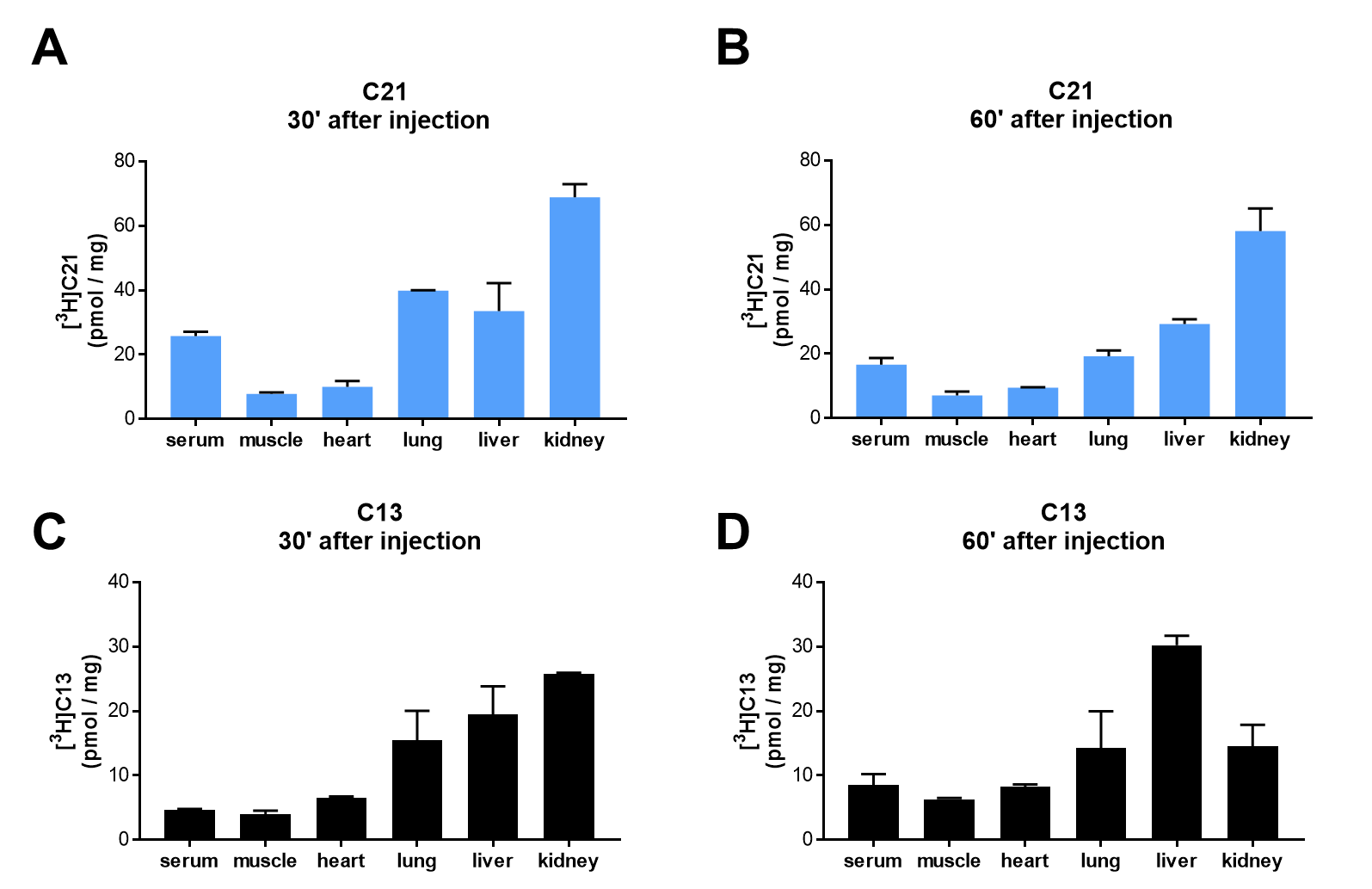


(**a, b**)**.** Concentration of [^3^H]Compound 21 (C21) in major organs 30 or 60 min after an intraperitoneal injection of 2 µCi/g of C21. (**c, d**)**.** Concentration of [^3^H]Compound 13 (C13) in major organs 30 or 60 min after an intraperitoneal injection of 2 µCi/g of C13. Data is shown as Mean ± SEM.

**Supplementary Figure 4.**

**Negative controls for the cell-based functional assays.**


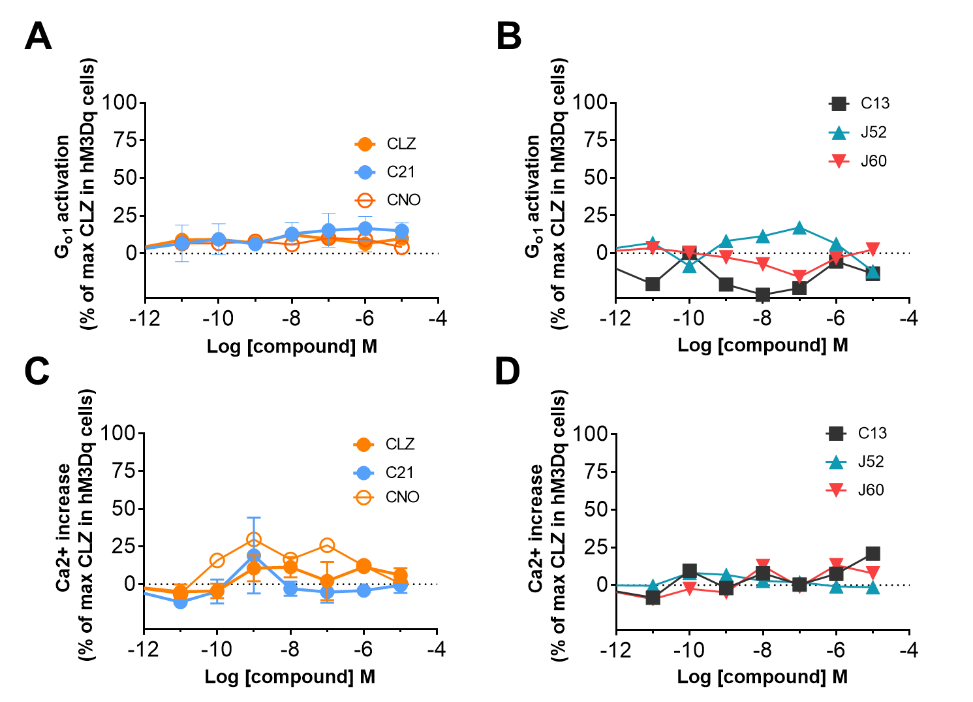


(**a, b**)**.** HEK-293 cells transfected with G_o_α-Rluc8, ß1 and γ2-mVenus but not DREADD did not show any functional response to Compound 21 (C21), clozapine (CLZ), CNO, Compound 13 (C13), JHU37152 (J52) or JHU37160 (J60). (**c, d**)**.** HEK-293 cells transfected with GCaMP6f but not DREADD did not show any functional response to C21, CLZ, CNO, C13, J52 or J60. Data is shown as Mean ± SEM.

**Supplementary Figure 5.**

**P-glycoprotein (P-gp) efflux assay.**


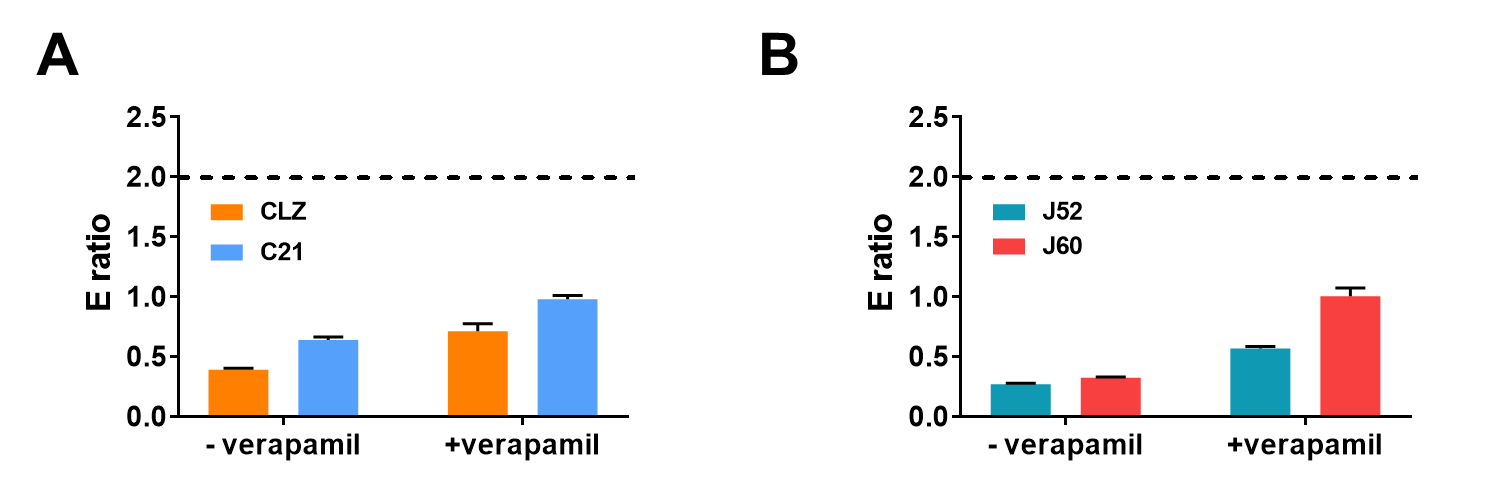


Efflux ratios for clozapine (CLZ), C21 (**a**), and JHU37152 (J52) and JHU37160 (J60) (**b**) in the P-gp assay. If a compound has an efflux ratio greater than two in the absence of verapamil, and verapamil reduces the efflux ratio, it indicates that the compound is a substrate of P-gp. Hence, none of these compounds is a substrate for P-gp. Data is shown as Mean ± SEM.

**Supplementary Figure 6.**

**JHU37160 bioanalytics and** **target engagement in monkeys.**


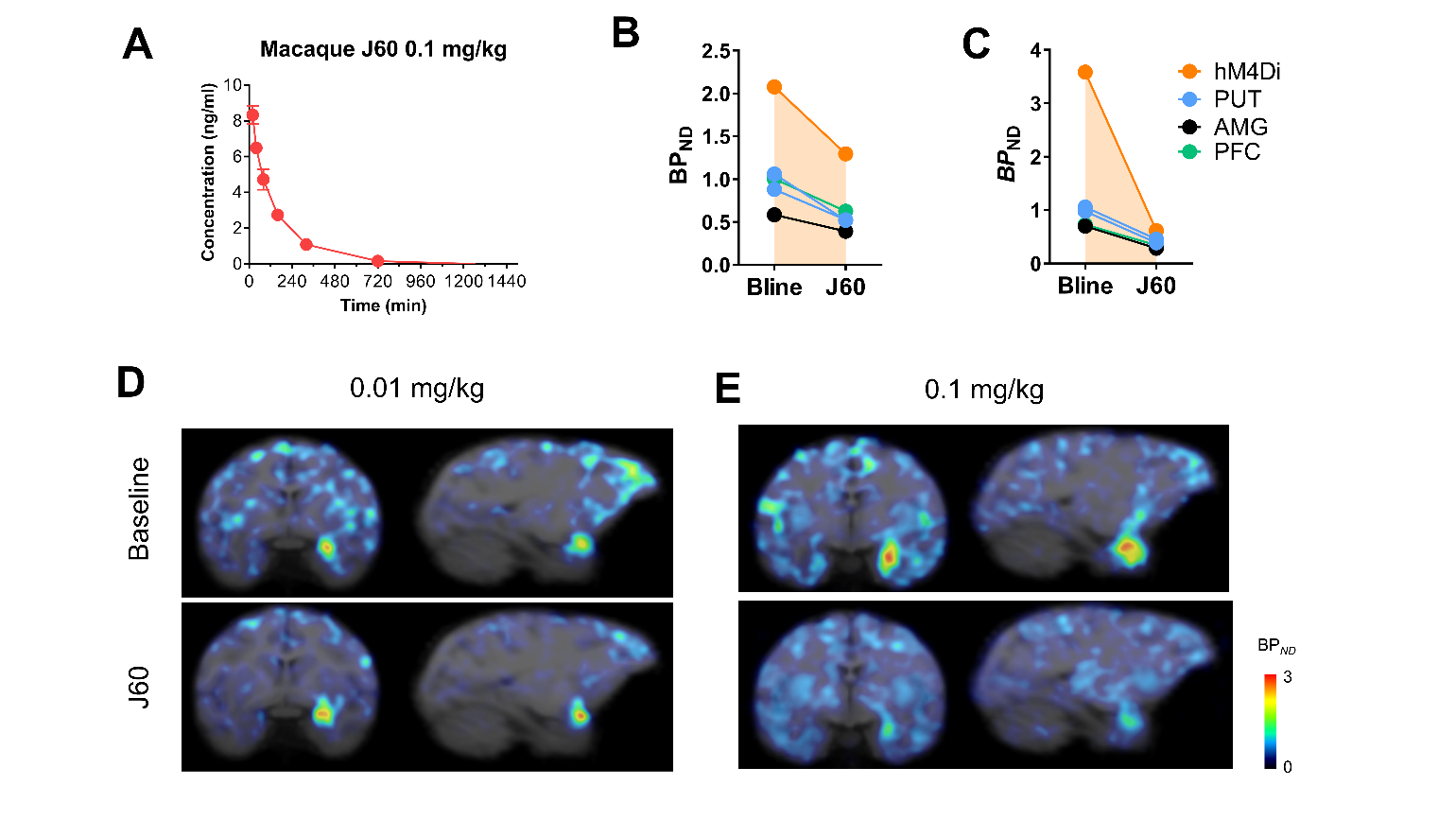


(**a**) JHU37160 concentration in rhesus macaque serum after a 0.1 mg/kg systemic dose. (**b-e**) J60 at 0.01 mg/kg (**b, d**) and 0.1 mg/kg (**c, e**) systemic dose blocks [^11^C]clozapine binding to hM4Di in two different monkeys Note: images in **e** are the full scale parametric maps of the image displayed in **Fig. 2G**. Data is shown as Mean ± SEM or individual values.

**Supplementary Figure 7.**

**Patterns of metabolic activation in WT mice.**


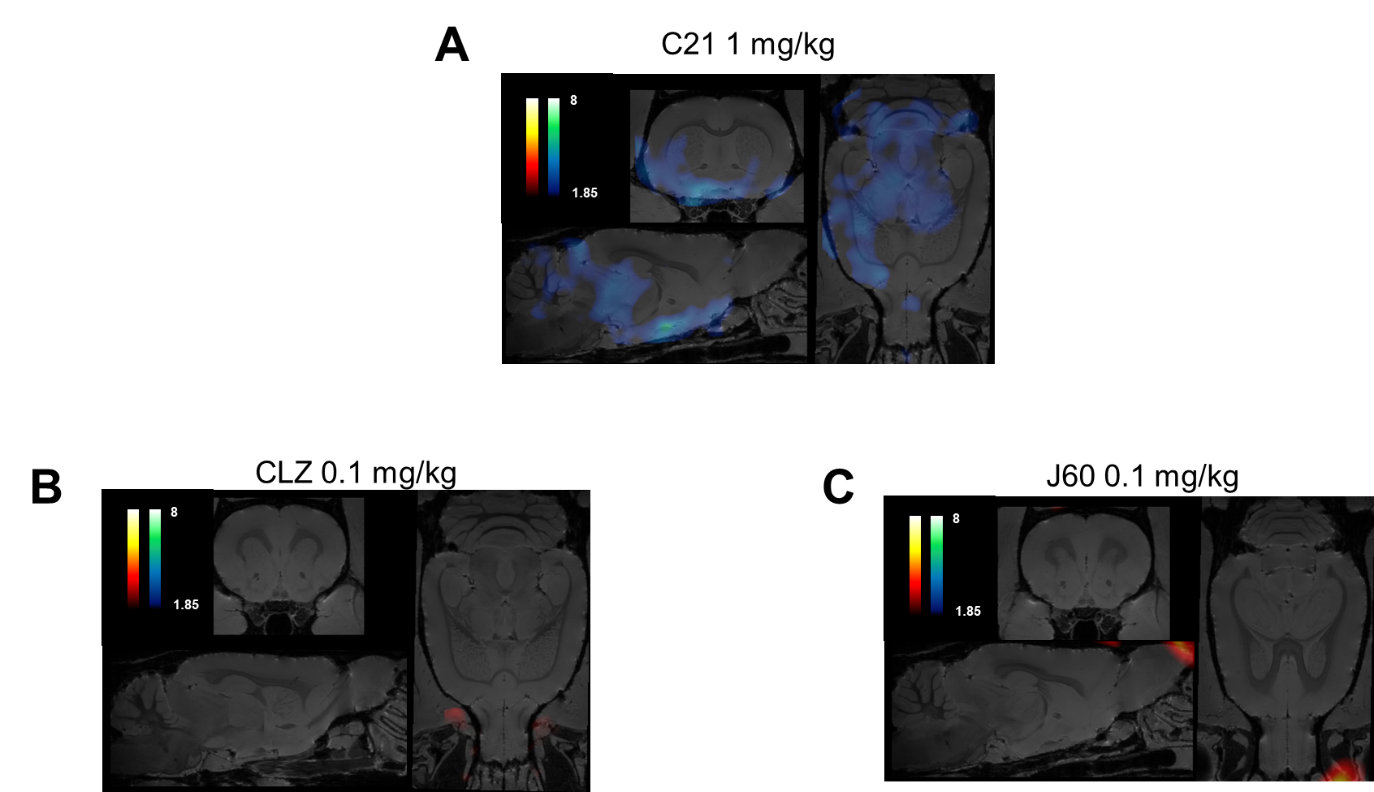


(**a**) Whole brain ^18^F-FDG metabolic mapping in WT mice shows decreased metabolic activity of multiple networks after a systemic administration of Compound 21 at 1 mg/kg. (**b, c**) Equipotent doses (0.1 mg/kg) of clozapine (CLZ) or JHU37160 (J60) did not show any significant changes in brain metabolic activity.

**Supplementary Figure 8.**

**Endogenous binding screens.**

**
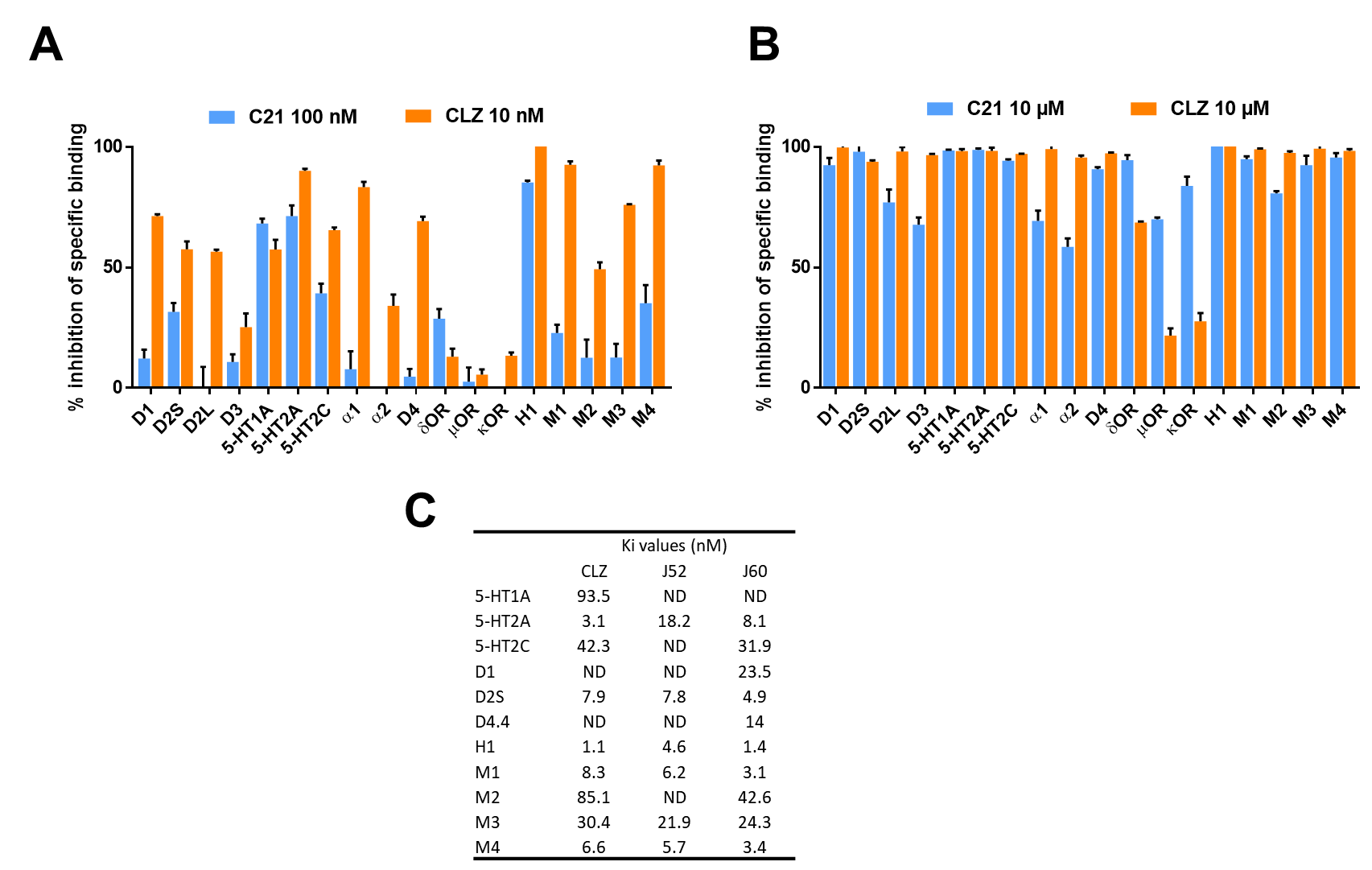
**

(**a**) Inhibition of specific binding at endogenous potential targets by Compound 21 (C21) or clozapine (CLZ). CLZ has an affinity of ~2 nM whereas C21 has an affinity of ~200 nM and at equipotent concentrations they show similar affinity profiles for endogenous receptors. (**b**) At higher concentrations (10 µM) they show specific binding to multiple targets. Importantly, C21 binds to opioid receptors but clozapine does not. (**c**) Ki values for CLZ, JHU27152 (J52) or JHU37160 (J60) for receptors at which a 100 nM concentration produced at least 50% of inhibition of the selective ligand specific binding. The three compounds show similar binding profiles at dopamine and muscarinic receptors but not at serotonin receptors. Given that clozapine acts as an antagonist or weak partial agonist at these targets, and that cell-based functional assays in HEK-293 cells endogenously expressing those receptors did not show any response to J52 and J60, (Supplementary Fig. 4) it is likely that J52 and J60 will behave as antagonists for the endogenous targets whereas they are potent agonists for DREADDs. data is shown as Mean ± SEM.

**Supplementary Figure 9.**

**Chrimson variant generation and characterization**


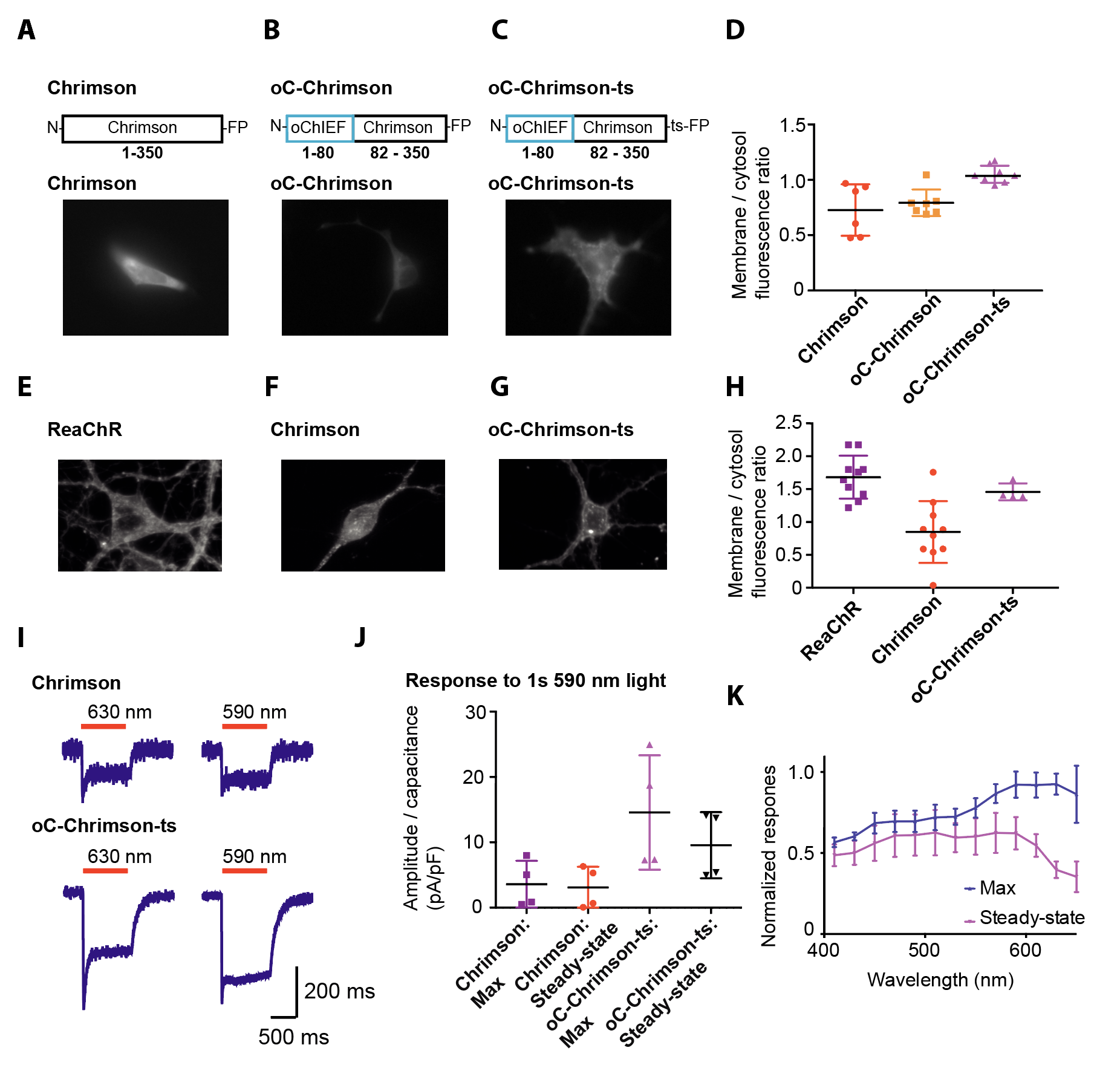


(**a**) The expression of Chrimson-FP construct in HEK293 cells show mostly cytosolic expression pattern. (**b**) oC-Chrimson is a chimeric protein of N-terminus of oChIEF (residues 1-80) and Chrimson (residues 82-350) has similar cytosolic expression pattern to Chrimson in HEK293 cells. The N-terminus of oChIEF was used as it contained a predicted signalling peptide sequence for membrane exporting and the utilization of the same N-terminus sequence in ReaChR and oChIEF resulted in superior membrane trafficking. (**c**) oC-Chrimson-ts had additional trafficking signal (amino acid sequence, KSRITSEGEYIPLDQIDINV) placed between Chrimson and FP that resulted in membrane trafficking with minimal cytosolic aggregation in HEK 293 cells. (**d**) oC-Chrimson-ts has the best membrane to cytosolic fluorescence ratio compared to Chrimson and oC-Chrimson in HEK293 cells. (**e-g**) The expression of ReaChR, Chrimson and oC-Chrimson-ts in cultured cortical neurons. ReaChR and oC-Chrimson-ts show comparable membrane expression with Chrimson expression mostly within the cytosol. (**h**) The quantification of membrane to cytosol fluorescence ratio in cultured neurons. (**i**) Example traces of voltage-clamp recording of 1 s light evoked Chrimson and oC-Chrimson-ts photocurrent in HEK293 cells at indicated wavelengths. In most cells, the Chrimson response is below 100 pA whereas the oC-Chrimson-ts photocurrents are typically greater (>200pA) when cells of comparable fluorescence are tested. (**j**) Quantification of the maximum and steady-state (measured at 950 – 1000 ms) photocurrent of Chrimson and oC-Chrimson-ts to 1 s 590 nm light. (**k**) The normalized photocurrent response of oC-Chrimson-ts from 650 nm to 400 nm (n = 3). Similar spectral response for Chrimson cannot be measured accurately due to the small size of photocurrent in most cells recorded. All graphs are shown as mean ± SD.

**Supplementary Figure 10.**

***In vivo* electrophysiological characterization of JHU37160.**


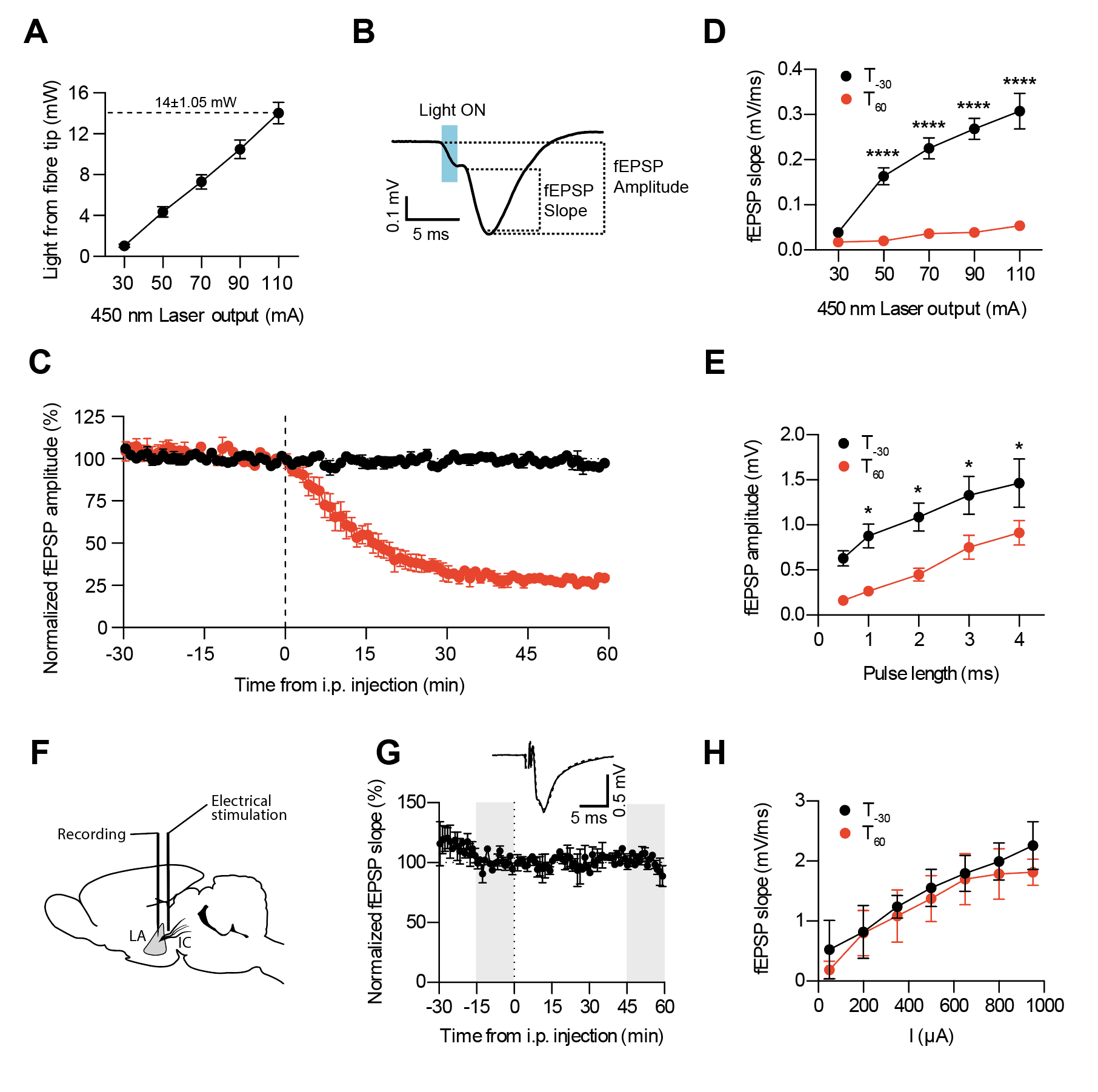


**(a)** Corresponding measurements of light intensity stimulation and measured light output from the tip of the optic fibre (n=8), showing a consistent light delivery through experiments. (**b**) Field EPSP measurements of slope and amplitude on a representative waveform. (**c**) fEPSP amplitude measurements corresponding to the same experiments presented in Figure3O, P (n=4). (**d**) Input-Output test curves for light intensity 30 min before (T_-30_) and 60 min after (T_60_) JHU37160 injection (n=4). 2-way ANOVA effect of power F (4, 30) = 21.09 and time F (1, 30) = 210.0, post-hoc Sidak’s multiple comparisons test shown as ****, p<0.0001 between T-30 and T60. (**e**) Input-Output test curves for pulse length 30 min before (T_-30_) and 60 min after (T_60_) JHU37160 injection (n=4). 2-way repeated measures ANOVA effect of pulse length F (4, 15) = 8.751 and time F (1, 15) = 47.06, post-hoc Sidak’s multiple comparisons test shown as *, p<0.05 between T-30 and T60. (**f**) Design of a control experiment with electrical stimulation of the Internal Capsule (IC) to LA pathway. (**g**) Time course of the effect of JHU37160 on non-hM4Di animals (n=2). Insert: representative waveforms from two selected time points (grey highlight in the graph) before and after infusion. (**h**) Input-Output test curves for stimulation intensity 30 min before (T_-30_) and 60 min after (T_60_) JHU37160 injection (n=2). 2-way repeated measures ANOVA effect of stimulation intensity F (6, 7) = 3.101 and time F (1, 7) = 8.445, post-hoc Sidak’s multiple comparisons test not significant for all intensities between T-30 and T60. In all panels, data is shown as Mean ± SEM.

**Supplemental Figure 11.**

**Radiometabolic analysis of [^18^F]JHU37107.**

**
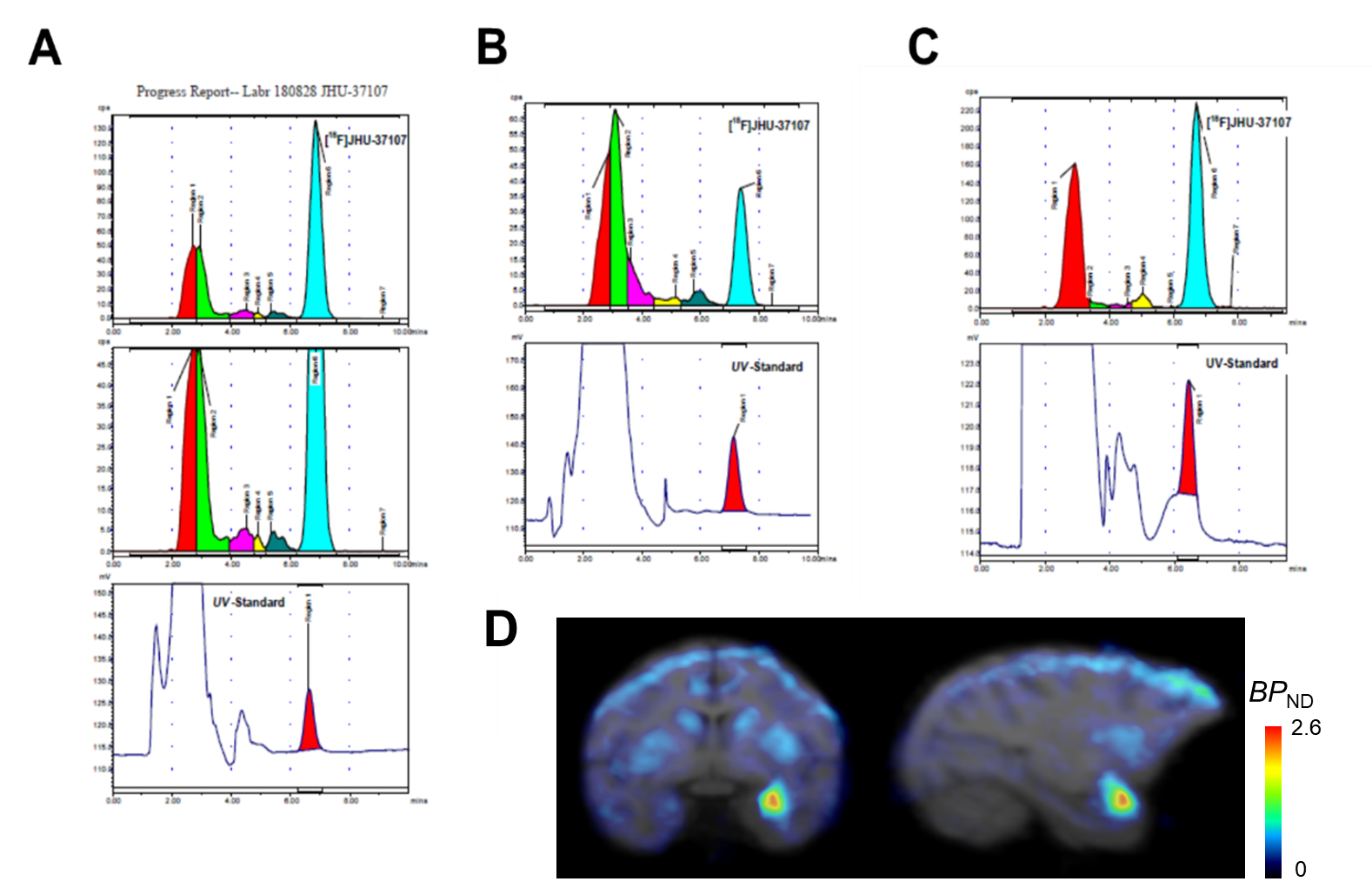
**

(**a**) Radiochromatographic profile of monkey plasma, at 30 min after the IV administration of [^18^F]JHU-37107 (J07). The parent radioactivity composition of plasma, region 6, was 56%. (**b**) Radiochromatographic profile of monkey plasma, at 180 min after the IV administration of J07. The parent radioactivity composition of plasma, region 6, was 24.2%. (**c**) Radiochromatographic profile of in vitro monkey whole blood (STD A), at 30 min of incubation with J07 at room temperature. The parent radioactivity composition of plasma, region 6, was 49.7%. **(d)** Full scale parametric map of the image displayed in Fig. 4M.

**Structural characterization of the novel described compounds**

**8-Chloro-11-(4-ethylpiperazin-1-yl)-2-fluoro-5*H*-dibenzo[*b*,*e*][1,4]diazepine** ( **JHU37107 )**

**^1^H NMR** (500 MHz, CDCl_3_) δ 7.07 (d, *J* = 2.3 Hz, 1H), 7.02 (td, *J* = 8.2, 2.9 Hz, 1H), 6.98 (dd, *J* = 8.7, 2.9 Hz, 1H), 6.84 (dd, *J* = 8.3, 2.4 Hz, 1H), 6.78 (dd, *J* = 8.6, 4.6 Hz, 1H), 6.62 (d, *J* = 8.3 Hz, 1H), 4.82 (s, 1H), 3.49 (bs, 4H), 2.55 (bs, 4H), 2.49 (dd, *J* = 14.4, 7.3 Hz, 2H), 1.13 (t, *J* = 7.2 Hz, 3H).

**^13^C NMR** (125 MHz, CDCl_3_) δ 171.3, 161.5, 159.8, 157.8, 148.6 (d, *J* = 2.2 Hz), 141.7, 140.5, 129.4, 127.0, 125.0 (d, *J* = 6.6 Hz), 123.5, 121.5 (d, *J* = 7.9 Hz), 120.2, 118.9 (d, *J* = 22.8 Hz), 116.6 (d, *J* = 23.5 Hz), 52.8, 52.5, 47.5, 12.1.

**HRMS** (ESI+) calculated for C_19_H_21_ClFN_4_ (M+H)^+^: 359.1439; found: 359.1435.

**HPLC**, Luna C18 (Phenomenex), 10 micron, 4.6*250 mm, flow rate 3 ml min^-1^, UV 254 nm, mobile phase CH_3_CN:water:trifloroacetic acid (230:770:1), RT = 12.5 min

**8-Chloro-11-(4-ethylpiperazin-1-yl)-4-fluoro-5*H*-dibenzo[*b*,*e*][1,4]diazepine** (**JHU37160**)

**^1^H NMR** (500 MHz, CDCl_3_) δ 7.15 – 7.09 (m, 1H), 7.07 (d, *J* = 2.3 Hz, 1H), 7.05 (d, *J* = 7.8 Hz, 1H), 6.95 (td, *J* = 8.0, 5.1 Hz, 1H), 6.85 (dd, *J* = 8.3, 2.4 Hz, 1H), 6.68 (d, *J* = 8.3 Hz, 1H), 5.34 (d, *J* = 3.8 Hz, 1H), 3.49 (bs, 4H), 2.55 (bs, 4H), 2.49 (q, *J* = 7.2 Hz, 2H), 1.13 (t, *J* = 7.2 Hz, 3H). **^13^C NMR** (125 MHz, CDCl_3_) δ 161.7, 154.5, 152.6, 141.8, 140.5 (d, *J* = 15.2 Hz), 140.0, 129.5, 127.1, 126.0, 125.59 (d, *J* = 3.7 Hz), 123.4, 123.1 (d, *J* = 7.5 Hz), 120.9, 117.4 (d, *J* = 20.0 Hz), 52.9, 52.6, 47.5, 12.1.

**HRMS** (ESI+) calculated for C_19_H_21_ClFN_4_ (M+H)^+^: 359.1439; found: 359.1456.

**HPLC**, Luna C18 (Phenomenex), 10 micron, 4.6*250 mm, flow rate 3 ml min^-1^, UV 254 nm, mobile phase CH_3_CN:water:trifloroacetic acid (230:770:1), RT = 11.3 min

**8-Chloro-11-(4-ethylpiperazin-1-yl)-1-fluoro-5*H*-dibenzo[*b*,*e*][1,4]diazepine** ( **JHU37152**)

**^1^H NMR** (500 MHz, CDCl_3_) δ 7.30 – 7.24 (m, 1H), 7.08 (s, 1H), 6.81 (d, *J* = 8.3 Hz, 1H), 6.77 (t, *J* = 9.0 Hz, 1H), 6.69 (d, *J* = 7.9 Hz, 1H), 6.64 (d, *J* = 8.3 Hz, 1H), 4.96 (s, 1H), 3.60 (bs, 4H), 2.61 (bs, 2H), 2.49 (dd, *J* = 13.8, 6.7 Hz, 4H), 1.14 (t, *J* = 7.2 Hz, 3H).

**^13^C NMR** (125 MHz, CDCl_3_) δ 160.6, 158.6, 156.2 (d, *J* = 2.1 Hz), 155.1 (d, *J* = 6.4 Hz), 142.4, 139.6, 156.2 (d, *J* = 2.1 Hz), 129.7, 126.4, 122.6, 120.3, 116.1, 112.04 (d, *J* = 15.8 Hz), 111.0 (d, *J* = 22.9 Hz), 52.9, 52.8, 52.5, 12.1.

**HRMS** (ESI+) calculated for C_19_H_21_ClFN_4_ (M+H)^+^: 359.1439; found: 359.1435.

**HPLC**, Luna C18 (Phenomenex), 10 micron, 4.6*250 mm, flow rate 3 ml min^-1^, UV 254 nm, mobile phase CH_3_CN:water:trifloroacetic acid (230:770:1), RT = 10.7 min
