# Supplementary material for: High-potency ligands for DREADD imaging and activation in rodents and monkeys
Source: Nat Commun. 2019 Oct 11;10:4627. doi: 10.1038/s41467-019-12236-z (PMC6788984; doi:10.1038/s41467-019-12236-z)
Supplement: Supplementary file 3 — Reporting Summary [file 41467_2019_12236_MOESM3_ESM.pdf]

nature research

Corresponding author(s): Michaelides, Richmond, Horti

Reporting Summary

Nature Research wishes to improve the reproducibility of the work that we publish. This form provides structure for consistency and transparency in reporting. For further information on Nature Research policies, see Authors & Referees and the Editorial Policy Checklist.

Statistical parameters

When statistical analyses are reported, confirm that the following items are present in the relevant location (e.g. figure legend, table legend, main text, or Methods section).

n/a	Confirmed	
☐	☒	The exact sample size (*n*) for each experimental group/condition, given as a discrete number and unit of measurement
☐	☒	An indication of whether measurements were taken from distinct samples or whether the same sample was measured repeatedly
☐	☒	The statistical test(s) used AND whether they are one- or two-sided<br>*Only common tests should be described solely by name; describe more complex techniques in the Methods section.*
☒	☐	A description of all covariates tested
☒	☐	A description of any assumptions or corrections, such as tests of normality and adjustment for multiple comparisons
☐	☒	A full description of the statistics including central tendency (e.g. means) or other basic estimates (e.g. regression coefficient) AND variation (e.g. standard deviation) or associated estimates of uncertainty (e.g. confidence intervals)
☐	☒	For null hypothesis testing, the test statistic (e.g. *F*, *t*, *r*) with confidence intervals, effect sizes, degrees of freedom and *P* value noted<br>*Give P values as exact values whenever suitable.*
☐	☐	For Bayesian analysis, information on the choice of priors and Markov chain Monte Carlo settings
☒	☐	For hierarchical and complex designs, identification of the appropriate level for tests and full reporting of outcomes
☒	☐	Estimates of effect sizes (e.g. Cohen's *d*, Pearson's *r*), indicating how they were calculated
☐	☒	Clearly defined error bars<br>*State explicitly what error bars represent (e.g. SD, SE, CI)*

Our web collection on statistics for biologists may be useful.

Software and code

Policy information about availability of computer code

Data collection

Provide a description of all commercial, open source and custom code used to collect the data in this study, specifying the version used OR state that no software was used.

Data analysis

Provide a description of all commercial, open source and custom code used to analyse the data in this study, specifying the version used OR state that no software was used.

For manuscripts utilizing custom algorithms or software that are central to the research but not yet described in published literature, software must be made available to editors/reviewers upon request. We strongly encourage code deposition in a community repository (e.g. GitHub). See the Nature Research guidelines for submitting code & software for further information.

Data

Policy information about availability of data

All manuscripts must include a data availability statement. This statement should provide the following information, where applicable:
- Accession codes, unique identifiers, or web links for publicly available datasets
- A list of figures that have associated raw data
- A description of any restrictions on data availability

All data is available in the main text or the supplementary materials.

Field-specific reporting

Please select the best fit for your research. If you are not sure, read the appropriate sections before making your selection.

☒ Life sciences ☐ Behavioural & social sciences ☐ Ecological, evolutionary & environmental sciences

For a reference copy of the document with all sections, see nature.com/authors/policies/ReportingSummary-flat.pdf

Life sciences study design

All studies must disclose on these points even when the disclosure is negative.

Sample size	Sample sizes were chosen based on our own results from previous experiments or reported data sets.
Data exclusions	No data were excluded from the analyses.
Replication	Behavioral experiments were replicated across different litters and time points. All attempts at replication were successful.
Randomization	Not relevant for this study as allocation of samples/organisms was not needed or intended.A
Blinding	Immunoluorescence and autoradiographic pictures were taken randomly from the slide without "searching" for a suitable area. For all other analyses experimenters were not blinded.

Reporting for specific materials, systems and methods

Materials & experimental systems

n/a	Involved in the study
☒	☐ Unique biological materials
☐	☒ Antibodies
☐	☒ Eukaryotic cell lines
☒	☐ Palaeontology
☐	☒ Animals and other organisms
☒	☐ Human research participants

Methods

n/a	Involved in the study
☒	☐ ChIP-seq
☒	☐ Flow cytometry
☒	☐ MRI-based neuroimaging

Antibodies

Antibodies used

Chicken anti-GFP
Supplier name: Abcam Inc.
Catalog #: ab13970
Clone name: polyclonal chicken
Lot #: GR236651-5

Rabbit anti-HA
Supplier name: Cell Signaling Technology
Catalog #: C29F4
Clone name: monoclonal rabbit
Lot #: 8

Goat anti-rabbit Alexa488
Supplier name: Invitrogen
Catalog #: A-11034
Clone name: polyclonal rabbit
Lot #: 1737902

Goat anti-chicken Alexa546
Supplier name: Invitrogen
Catalog #: A-11040
Clone name: polyclonal chicken
Lot #: 1830310

Rabbit anti-mCherry
Supplier name: Abcam Inc.
Catalog #: ab167453
Clone name: polyclonal rabbit
Lot #: GR312817-7

Goat anti-rabbit Alex647
Supplier name: Invitrogen
Catalog #: A-21245
Clone name: polyclonal rabbit
Lot #: 1729791

Goat anti-chicken Alexa488
Supplier name: Invitrogen
Catalog #: A-11039
Clone name: polyclonal chicken
Lot #: 1691381

Validation

Mitew S. et al., Pharmacogenetic stimulation of neuronal activity increases myelination in an axon-specific manner. Nature communications 9:306 (2018).

Sun X. et al., Distinct multilevel misregulations of Parkin and PINK1 revealed in cell and animal models of TDP-43 proteinopathy. Cell Death & Disease 9:953 (2018).

Brendel M. et al., Increase of TREM2 during Aging of an Alzheimer's Disease Mouse Model Is Paralleled by Microglial Activation and Amyloidosis. Frontier Aging Neuroscience 9:8 (2017).

Wang A.C. et al., Loss of O-GlcNAc glycosylation in forebrain excitatory neurons induces neurodegeneration. Proc Natl Acad Sci USA 113(52): 15120-15125 (2016).

Gascón S. et al., Transcription of the NR1 subunit of the N-methyl-D-aspartate receptor is down-regulated by excitotoxic stimulation and cerebral ischemia. The Journal of Biological Chemistry 280(41): 35018-35027 (2005).

Qamar S. et al., FUS Phase Separation Is Modulated by a Molecular Chaperone and Methylation of Arginine Cation-p Interactions. Cell 173:720-734.e15 (2018).

Miller A.P. et al., Acute death of astrocytes in blast-exposed rat organotypic hippocampal slice cultures. PloS One 12(3):e0173167 (2017).

Franklin T.B. et al., Prefrontal cortical control of a brainstem social behavior circuit. Nature neuroscience 20(2): 260-270 (2017).

Eukaryotic cell lines

Policy information about cell lines

Cell line source(s): Human HEK293 cells were obtained from ATCC (CRL-1573)

Authentication: N/A

Mycoplasma contamination: Cells were routinely tested for mycoplasma contamination using Mycoalert Mycoplasma detection it from Lonza,

Commonly misidentified lines (See ICLAC register): N/A

Animals and other organisms

Policy information about studies involving animals; ARRIVE guidelines recommended for reporting animal research

Laboratory animals

Wild-type mice (C57BL/6J) were ordered from Jackson Laboratories and rats (Sprague-Dawley) were ordered from Charles River. Rodents were male and ordered at ~6-weeks of age. Transgenic mice were bred at NIDA breeding facility. Transgenic mice expressing the enzyme cre recombinase under the control of the dopamine D1 receptor promoter (D1-Cre, FK150 line, C57BL/6J congenic, Gensat, RRID: MMRRC_036916-UCD) were crossed with transgenic mice with cre recombinase-inducible expression of hM4Di DREADD (R26-hM4Di/mCitrine, Jackson Laboratory, stock no. 026219) or hM3Dq DREADD (R26-hM3Dq/mCitrine, Jackson Laboratory, stock no. 026220). Three male rhesus monkeys (Macaca mulatta) weighed 8 – 12 kg. All experiments and procedures followed NIH guidelines and were approved by each institute's animal care and use committees.

Wild animals

Provide details on animals observed in or captured in the field; report species, sex and age where possible. Describe how animals were caught and transported and what happened to captive animals after the study (if killed, explain why and describe method; if released, say where and when) OR state that the study did not involve wild animals.

Field-collected samples

For laboratory work with field-collected samples, describe all relevant parameters such as housing, maintenance, temperature, photoperiod and end-of-experiment protocol OR state that the study did not involve samples collected from the field.
